# The National Medical Dictionary

**Published:** 1890-03

**Authors:** 


**The National Medical Dictionary.** Including English, French, German, Italian and Latin Technical Terms used in Medicine and the Collateral Sciences, and a Series of Tables of Useful Data. By JOHN S. BILLINGS, A. M., M. D., LL. D., Edin. and Harv., D. C. L., Oxon. With colaboration of W. O. Atwater, M. D., Frank Baker, M. D., S. M. Burnett, M. D., W. T. Councilman, M. D., James M. Flint, M. D., J. A. Kidder, M. D., William Lee, M. D., R. Lorini, M. D., Washington Matthews, M. D., C. S. Minot, M. D., and H. C. Yarrow, M. D. In two volumes. Vol. I., A to J. Vol. II., K to Z. Philadelphia: Lea Brothers & Co. 1890.

To review a dictionary, in the ordinary sense of the term, is not only unnecessary, but it is next to impossible. Nevertheless, such a work as that before us deserves to be "noticed" in the fullest application of the word.

For many years the literature of medicine has suffered in the department of lexicography. Dunglison's Dictionary, so long the standard, has not been able to meet the demands of the rapid-growing nomenclature, and is fast becoming a "back number." Foster is engaged in preparing a comprehensive work that will meet the demands of the period, but, unfortunately, only one volume has yet been put forth, and the remaining ones are painfully slow in appearing. Hence, it is with pleasure we greet this work of Billings that comes forth so opportunely, with its 84,844 words and phrases, including 25,496 from the Latin, 9,158 from the French, 16,708 from the German, and 6,514 from the Italian, besides synonyms from the three latter languages, given only in connection with the English or Latin primes.

Then there are prefixed to the first volume a series of tables convenient and useful for reference. These include a table of doses; of antidotes; of the inch and metre system of measuring spectacles; of thermometric scales; of the dimensions of the fetus at different ages; of the average dimensions of the parts and organs of the adult human body, and weights of the organs; percentage of nutritive ingredients in various foods; and a table of life expectations as derived from the records of American life insurance companies.

But it is as an authority on words and terms used in medicine that the work is to be especially commended. Here it will be found invaluable, and must supplant every other authority extant, excepting Foster's, though it cannot be considered as in competition with him. It would appear from such examination of this dictionary as we have been able to make, that it must become an indispensable part of a physician's library; while dentists, anthropologists, teachers, sanitarians, and all interested in the study of the collateral sciences, cannot well afford to deny themselves the convenience of its possession. The two quarto volumes contain pp. xliv.—1,530, double columned, printed on No. 1 book paper, in plain type, and are most perfect specimens of the book-makers' art.

The author, who is concededly the greatest living medical bibliographer, in the creation of this work takes rank as one of the greatest lexicographers of the day. These two special fields in which he has conspicuously distinguished himself, complement each other in a most gratifying and happy manner, and the name of Billings must henceforth and forever adorn the historical pages of American medicine. It is, and ought to be, the pride of the medical staff of the United States Army, that it can contribute such an ornament to the guild.

